# The different biological effects of single, fractionated and continuous low dose rate irradiation on CL187 colorectal cancer cells

**DOI:** 10.1186/1748-717X-8-196

**Published:** 2013-08-09

**Authors:** Hao Wang, Jinna Li, Ang Qu, Jingjia Liu, Yong Zhao, Junjie Wang

**Affiliations:** 1 Department of Radiation Oncology, Peking University Third Hospital, North Road No. 49, Haidian District, 100191, Beijing, China; 2 Transplantation Biology Research Division, State Key Laboratory of Biomembrane and Membrane Biotechnology, Institute of Zoology, Chinese Academy of Sciences, Beijing, China

**Keywords:** ^125^I seeds, CL187 cell, DNA repair

## Abstract

**Purpose:**

To determine the biological effectiveness of single, fractionated and continuous low dose rate irradiation on the human colorectal cancer cell line CL187 in vitro and explore the cellular mechanisms.

**Materials and methods:**

The CL187 cells were exposed to radiation of 6 MV X-ray at a high dose rate of 4Gy/min and ^125^I seed at a low dose rate of 2.77 cGy/h. Three groups were employed: single dose radiation group (SDR), fractionated dose radiation group (FDR) by 2Gy/f and continuous low dose rate radiation group (CLDR). Four radiation doses 2, 4, 6 and 8Gy were chosen and cells without irradiation as the control. The responses of CL187 cells to distinct modes of radiation were evaluated by the colony-forming assay, cell cycle progression as well as apoptosis analysis. In addition, we detected the expression patterns of DNA-PKcs, Ku70 and Ku80 by Western blotting.

**Results:**

The relative biological effect for ^125^I seeds compared with 6 MV X-ray was 1.42. 48 hrs after 4Gy irradiation, the difference between proportions of cells at G2/M phase of SDR and CLDR groups were statistically significant (p = 0.026), so as the FDR and CLDR groups (p = 0.005). 48 hrs after 4Gy irradiation, the early apoptotic rate of CLDR group was remarkably higher than SDR and FDR groups (CLDR vs. SDR, p = 0.001; CLDR vs. FDR, p = 0.02), whereas the late apoptotic rate of CLDR group increased significantly compared with SDR and FDR group (CLDR vs. SDR, p = 0.004; CLDR vs. FDR, p = 0.007). Moreover, DNA-PKcs and Ku70 expression levels in CLDR-treated cells decreased compared with SDR and FDR groups.

**Conclusions:**

Compared with the X-ray high dose rate irradiation, ^125^I seeds CLDR showed more effective induction of cell apoptosis and G_2_/M cell cycle arrest. Furthermore, ^125^I seeds CLDR could impair the DNA repair capability by down-regulating DNA-PKcs and Ku70 expression.

## Introduction

Colorectal cancer is one of the leading causes of death in the world, and more than 170,000 new patients are diagnosed in China each year. In general, rectal cancer has a relatively higher risk of recurrence. Once the rectal cancer recurred, regular treating strategies such as operation, chemotherapy and external beam radiotherapy failed to achieve appreciative outcomes [1,2]. For those patients who could not receive an operation and re-irradiation, there were few strategies to control the disease progression, and their median survival time was between 3 and 6 months [2,3]. Locally recurrent rectal cancer (LRRC) remained a challenging problem in clinics, due to poor survival and severe symptoms such as pain and bleeding. Image guided permanent implantation of radioactive ^125^I seeds into the tumor site shows major advantages of delivering a high dose of irradiation to the tumor with a very sharp fall-off outside the implanted volume. In this regard, ^125^I seed permanent implantation has been recommended by the National Comprehensive Cancer Network to treat patients with low and intermediate risk prostate cancer [4,5]. ^125^I seed permanent implantation was also employed to treat pancreatic cancer and head and neck neoplasm because of its unique properties of locally limited irradiation [6,7]. Several clinical studies confirmed that ^125^I seed implantation was feasible, effective and safe as a salvage or palliative treatment for those patients suffering recurrent and re-recurrent rectal cancer with minimally invasion [8,9]. Although there are several reports on the biological effects of continuous low dose rate radiation, the different effectiveness of variant dose rate irradiation on the human colorectal cancer cell line and the relevant cellular mechanisms are still largely unidentified. The aim of the present study is to determine the direct biological effectiveness of single dose radiation (SDR), fractionated dose radiation (FDR) and continuous low dose rate radiation (CLDR) on human colorectal cancer cell line CL187 cultured in vitro, and we also aimed to unravel the underlying cellular and molecular mechanisms behind distinct modes of irradiation.

## Materials and methods

### Reagents

RPMI 1640 media for cell culture was purchased from Gibco (Life Technologies Corporation, NY, USA). Propidium Iodide (PI) was purchased from Cell Signaling Company (Cell Signaling Technology, Beverly, MA, USA). Anti-DNA-PKcs, anti-Ku80 and anti-Ku70 antibodies were obtained from Cell Signaling Technology, Inc (Cell Signaling Technology, MA, USA).

### Cell lines and cell culture

The human colorectal cancer cell line, CL187, was kindly gifted by the Beijing Institute for Cancer Research [10]. In brief, CL187 cells are maintained in RPMI 1640 supplemented with 100 IU/ml penicillin, 100 mg/ml streptomycin, 4 mM glutamine, and 10% heat-inactivated fetal bovine serum (Hangzhou Sijiqing Biological Engineering Materials Company, China) in a humidified atmosphere of 95% air and 5% CO_2_ at 37°C. The medium was replaced every two or three days.

### ^125^I seeds and X-ray irradiation

**Figure 1:**
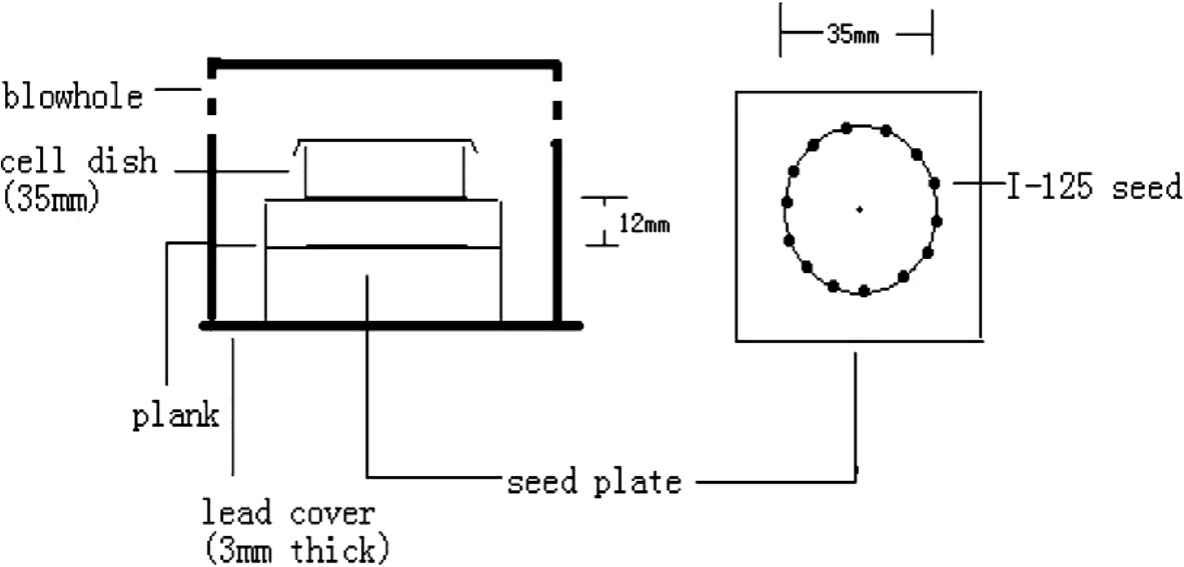
**Schematic representation of an in vitro model for iodine-125 seed irradiation.** The model was consisted of a lower seed plate layer and an upper-cell culture plate layer. In the seed plate, 14 seeds with the same activity were equally spaced within recesses around a 35-mm diameter circumference. For the cell-culture plate, similar recesses were made around a 35-mm diameter circumference. The height between the seed plate and the bottom of the Petri dish was 12 mm.

An in-house model for in vitro iodine-125 seed irradiation (shown in Figure 1) was developed for this study [10-12]. The model consisted of a 3-mm-thick polystyrene panel, with a lower seed plate layer and an upper-cell culture plate layer. In the seed plate, 14 seeds with the same activity were equally spaced within recesses (4.5 × 0.8 mm) around a 35-mm diameter (D) circumference. For the cell-culture plate, similar recesses were made around a 35-mm D circumference. The center of the cell-culture plate was vertically aligned above the seed plate, so that a 35-mm Petri dish could be placed on it during the experiment. The height (H) between the seed plate and the bottom of the Petri dish was 12 mm, with a D/H ratio of 2.9. The purpose of this design was to obtain a relatively homogeneous dose distribution at the bottom of the Petri dish. The polystyrene assembly was encased in a 3-mm-thick lead chamber with a vent hole, so that the whole model could be kept in the incubator during the study. The incubator played a protective role by maintaining constant cell-culture conditions. Model 6711 ^125^I seeds were provided by the Ningbo Junan Pharmaceutical Technology Company (Ningbo, Zhe Jiang province, China). The activity of the single seed used in this study was 92.5 MBq (2.5 mCi), which translates to an initial dose rate of 2.77 cGy/h to model cells. The dose uniformity of the irradiation model in the cell plane was 1.34, which was in agreement with previous study [13]. This model was thus validated by using thermoluminescent dosimetry (TLD) measurement and the absorbed doses for various exposure times, in various culture planes, were also measured and verified. The exposure times for delivering doses of 2, 4, 6 and 8Gy were 73.7, 154.6, 245.8 and 345.1 hours (i.e., 3.07, 6.44, 10.24 and 14.38 days), respectively [10-12]. Liner accelerators producing 6 MV X-ray beams were provided by the 306^th^ Hospital of the People’s Liberation Army (Beijing, China). The dose rate was 400 cGy/min and the source-to-skin distance (SSD) was 100 cm. The surface of the culture dishes was covered by 1.5 cm thickness packing materials. Radiation dose were 0, 2, 4, 6 and 8Gy. The absorbed dose was equivalent to CLDR treatment.

### Cell death assay

Exponentially-growing CL187 cells was left untreated or exposed to ^125^I seeds and X-ray at the absorbed doses of 2, 4, 6 and 8 Gy, respectively. After irradiation, cells were cultured for another 24 or 48 hrs, followed by cell harvest and stained with trypan blue. The percentage of dead cells was counted for each delivered dose as: number of dead cells/total number of cells collected × 100%. The mean was calculated from three parallel samples for each dose point.

### Clonogenic survival assay

Briefly, exponentially growing cells were exposed to different dosages (0, 2, 4, 6 and 8 Gy) with SDR, FDR or CLDR. After further culture for 14 days, colonies were fixed with methanol and stained with Giemsa. Colonies with more than 50 cells were manually counted. The plating efficiency (PE) and survival fraction (SF) were calculated as follows: PE = (colony number/inoculating cell number) × 100%. SF = PE (tested group)/PE (0-Gy group) × 100%. The SF_2_ of different groups were used to calculate the relative biological effect (RBE). RBE = SF_2(CLDR)_/ SF_2(SDR/FDR)_.

### Cell cycle and apoptosis analysis

Cells were harvested 24 and 48 hrs after SDR, FDR or CLDR. Cells were counted and washed twice with cold PBS. For the preparation of the cells, an apoptosis assay by Beyotime Biotech, Jiangsu, China, based on AnnexinV and PI staining was used. The cells were prepared according to the manufactures instructions, and the stained cells were manufactured with Beckman Coulter Epics XL as soon as possible. Cells used for cell-cycle analysis were fixed with cold 70% ethanol and stained with PI after RNase treatment and analyzed by fluorescence-activated cell sorting (FACS) using BD Calibur and ModFit software (Verity Software House, Topsham, MN). Three independent experiments were conducted for each group.

### Immunoblotting and antibodies

Proteins were extracted from cells of different groups 30 minutes and 6 hours after radiation [14], cells without radiation served as the control. The protein concetration was determined by Bicinchoninic Acid (BCA) protein assay (Beyotime Biotech, China). Cell lysates containing 20 μg of protein were resolved on sodium dodecyl-sulfate polyacrylamide gel electrophoresis (SDS-PAGE) and transferred to PVDF membranes (Hybond-P, Amersham, Buckinghamshire, UK). The membranes were blocked with TBS containing 0.1% Tween-20 (TBST) containing 5% non-fat milk for 2 hrs at room temperature and then incubated overnight at 4°C with the primary antibodies. Membranes were washed three times with TBST, and then incubated with second antibody for 2 hrs at room temperature. After washing with TBST, proteins were detected by enhanced chemiluminescence (ECL) detection kit (Pierce Biotechnology). Antibodies for immunoblotting in this study were as follows: anti-DNA-PK, anti-Ku80, anti-Ku70 (Cell Signaling Biotechnology, Beverly, MA, USA), anti-actin (Santa Cruz Biotechnology, Santa Cruz, CA), anti-rabbit IgG-HRP and anti-mouse IgG-HRP (Santa Cruz Biotechnology, Santa Cruz, CA).

### Statistical analysis

The statistical analysis was performed with SPSS (version 13.0; SPSS Inc., Chicago, IL). Measurement data are presented as the mean ± standard deviation (x ± SD). The difference between the means of different groups was analyzed with a pair-designed t-test, a value of p < 0.05 was considered significant.

## Results

### CLDR inhibited survival of CL187 cells more efficiently than SDR and FDR

**Figure 2:**
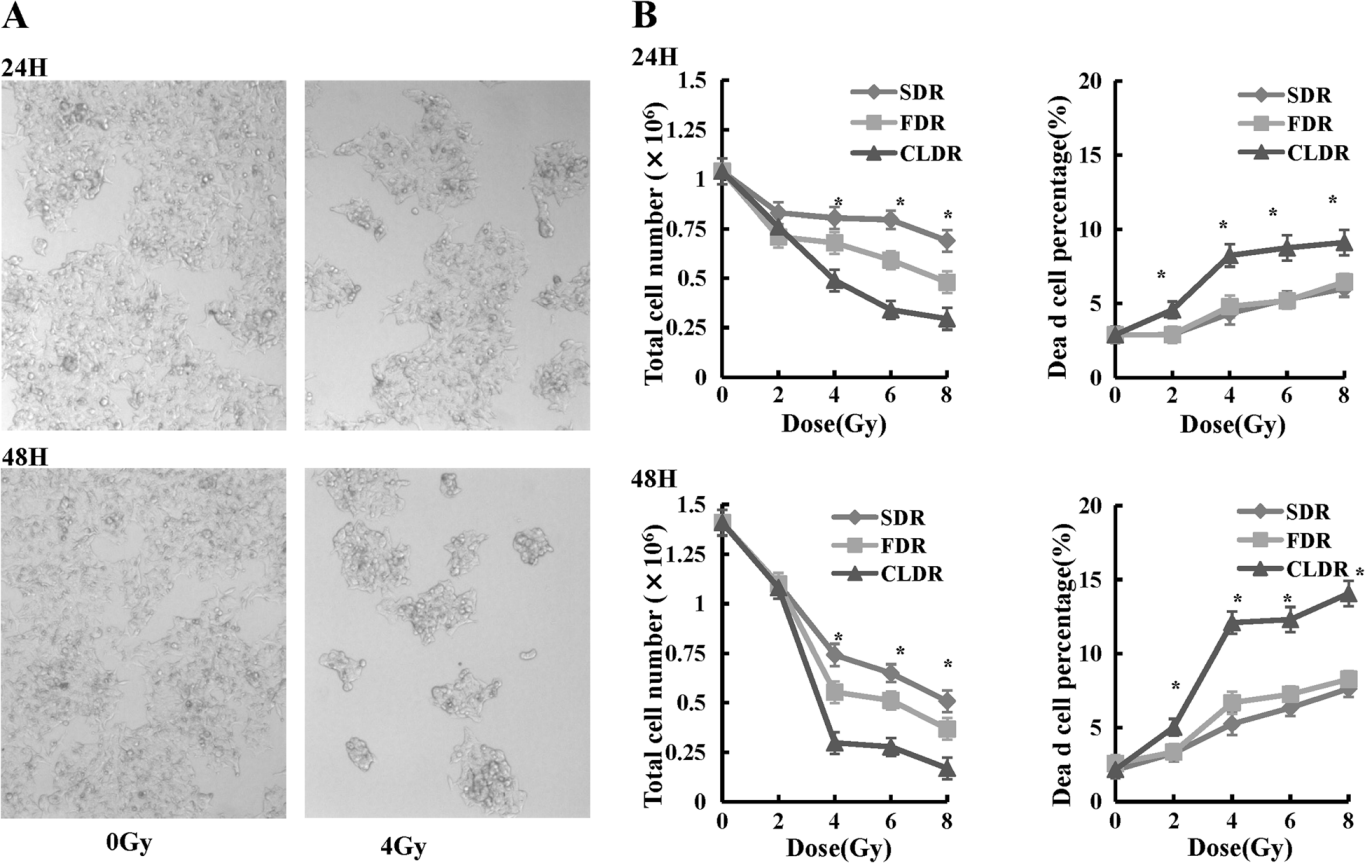
**The effects of SDR, FDR and CLDR on cell proliferative ability and cell death. A**. Cell morphological changes 24 and 48 hrs after CLDR (4Gy), and it is obvious that cell layers are less condensed in CLDR group. **B**. The inhibitory effects of irradiation on cell proliferation and cell death. Statistical analysis to show the changes of total cell number and the frequency of dead cells of three groups at 24 and 48 hrs along 0, 2, 4, 6 and 8Gy irradiation. One representative of three independent experiments with identical results was shown.*p < 0.05. (show * in figures).

We firstly determined the effect of CLDR on CL187 cell survival. As shown in Figure 2A, it is obvious that there are less cells both 24 and 48 hrs after CLDR treatment (4Gy). In order to evaluate the direct effects of SDR, FDR and CLDR on cell survival and cell death, we counted the total cell number and dead cell number 24 and 48 hrs after irradiation. Our data showed that the total cell number of CL187 cells decreased and the proportion of dead cells increased accordingly at 24 and 48 hrs after 2, 4, 6 and 8Gy irradiation (Figure 2B). Importantly, at the same dose, the CLDR resulted in increased percentages of cell death and thus the total cell numbers decreased more significantly, especially 48 hrs after irradiation compared with SDR and FDR. The difference of total cell number between SDR and CLDR groups was statistically significant (t = 34.28, p = 0.001), so as the FDR and CLDR groups (t = 29.48, p = 0.001) 48 hrs after 4Gy irradiation. In the meantime, the difference of the dead cell percentage between SDR and CLDR groups was statistically significant (t = -12.38, p = 0.006), so as the FDR and CLDR groups (t = -24.61, p = 0.002).

### Clonogenic survival of CL187 cells treated with different fraction model of irradiation

**Figure 3:**
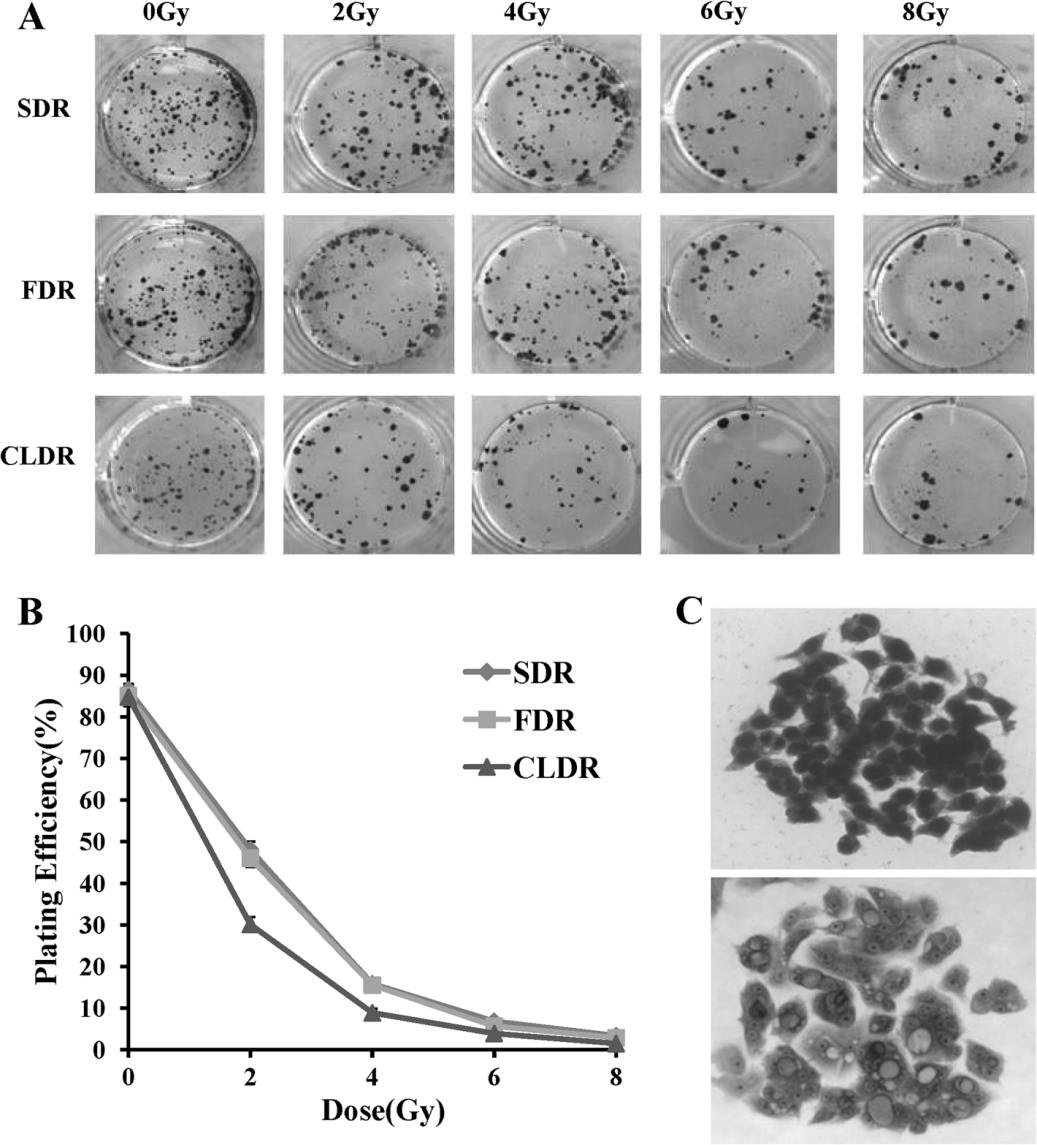
**Colony forming assay of CL187 cells after SDR, FDR and CLDR. A**. The inhibitory effects of irradiation on cell colony formation. The clones of the SDR, FDR and CLDR group after 0, 2, 4, 6 and 8Gy irradiation. Representative dishes after colony-forming assay are shown. **B**. The curves to show the plating efficiency of SDR, FDR and CLDR group at different dose points. **C**. Cell morphology of a clone before (upper) and after (lower) radiation.

To further confirm the superiority of CLDR, we performed colony forming assay, which was defined as the ability to maintain clonogenic and to form colonies. Our data showed that cell-killing effects were closely correlated to the dose rate of radiation. The plating efficiency of CL187 cells after different dose rate irradiation is shown in Figure 3. At the same doses, the survival fractions of CL187 cells treated with CLDR were always lower than CL187 cells treated with SDR or FDR especially with 2 or 4Gy irradiation, whereas high dose treatments could have the same cell-killing effects. The plating efficiency of 0, 2, 4, 6 and 8Gy of SDR group was 86.51 ± 1.53%, 48.15 ± 2.25%, 15.84 ± 1.02%, 6.75 ± 0.62 and 3.37 ± 0.39%, respectively. The plating efficiency of FDR group at 0, 2, 4, 6 and 8Gy was 85.12 ± 2.56%, 46.03 ± 1.95%, 15.52 ± 0.92%, 6.87 ± 0.69% and 2.73 ± 0.39%, respectively. And the plating efficiency of CLDR group was 84.84 ± 2.15%, 32.41 ± 1.81%, 8.80 ± 0.92%, 3.83 ± 0.52% and 1.43 ± 0.35%, respectively. In the present study, the survival fraction of CL187 cells after 2 Gy irradiation of SDR, FDR and CLDR group was 0.55, 0.54 and 0.38, respectively. The estimated relative biological effect for ^125^I seeds compared with 6 MV X-ray was 1.42.

### CLDR-treated CL187 cells displayed severe G_2_/M arrest

**Figure 4:**
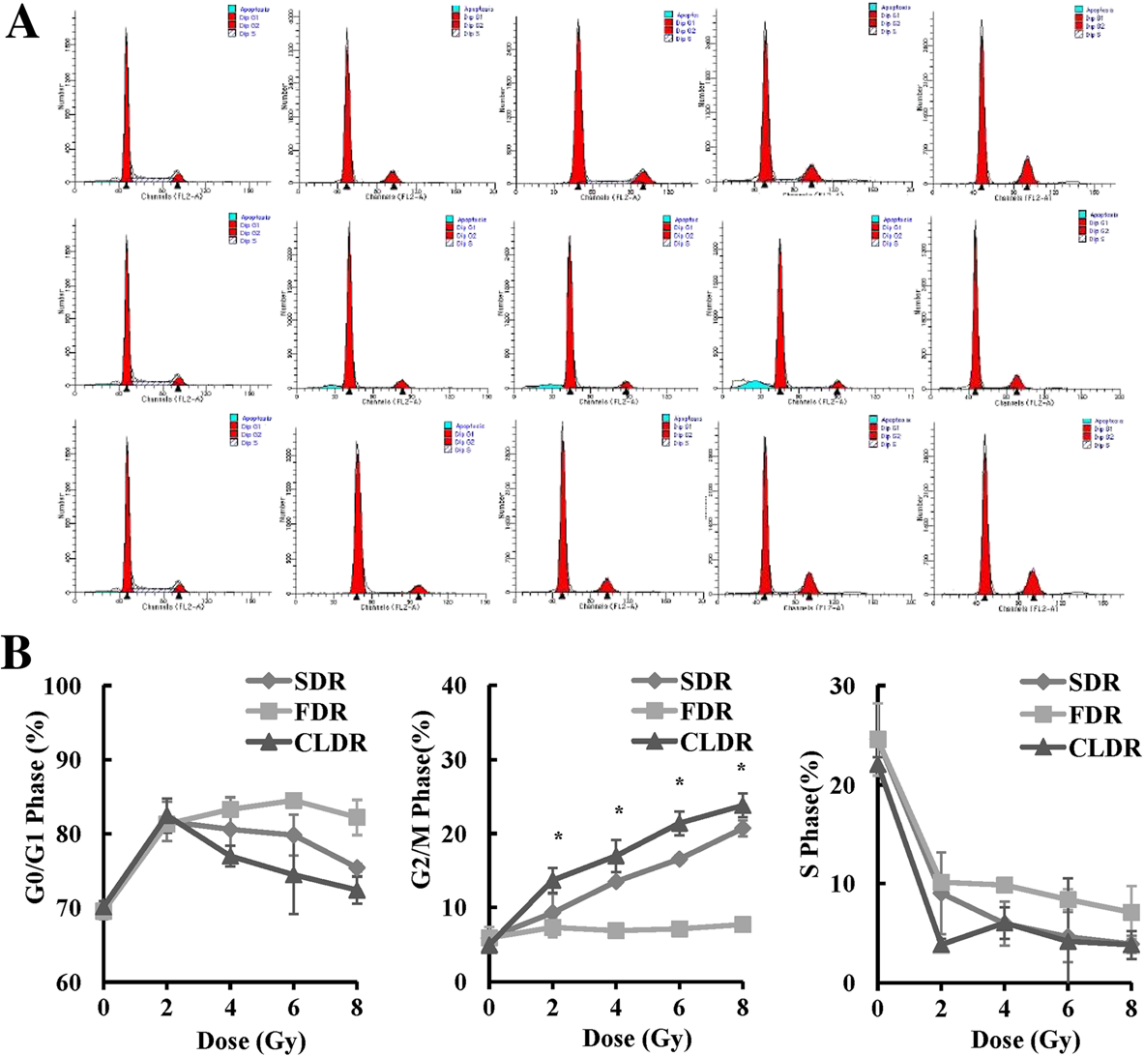
**Cell cycle analysis after different modes of irradiation. A**. FACS analysis of cell cycle distribution of CL187 cells upon distinct modes of irradiation. Representative profiles of cell cycle kinetics were shown 48 hrs after 0, 2, 4, 6 and 8 Gy irradiation. CL187 cells were fixed, permeablized, and stained with PI to measure DNA content by FACS analysis. The percentages of cells in each phase of the cell cycle determined by their DNA contents are indicated **B**. Statistical analysis showed the percentages of CL187 cells at G0/G1, G2/M or S phase after SDR, FDR or CLDR. CLDR caused increased G2/M phase. One representative of three independent experiments with identical results was shown. *p < 0.05. (show * in figures).

To determine the influence of different modes of irradiation on cell cycle progression, we detected the cell cycle distribution 48 hrs after 0, 2, 4, 6 and 8Gy irradiation. As shown in Figure 4, all three groups turned out to show the G_2_/M phase arrest, and it was interesting that increased percentages of cells arrested at G_2_/M phase occurred in CLDR group. The proportions of cells at G_2_/M phase of 0, 2, 4, 6 and 8Gy in CLDR group were 4.92 ± 0.02%, 13.67 ± 1.67%, 16.41 ± 2.19%, 21.40 ± 1.61% and 23.81 ± 1.61%, respectively. 48 hrs after 4Gy irradiation, the difference between proportions of cells at G_2_/M phase of SDR and CLDR groups were statistically significant (t = -6.03, p = 0.026), so as the FDR and CLDR groups (t = -13.98, p = 0.005).

### CLDR leads to increased apoptosis of CL187 cells

**Figure 5:**
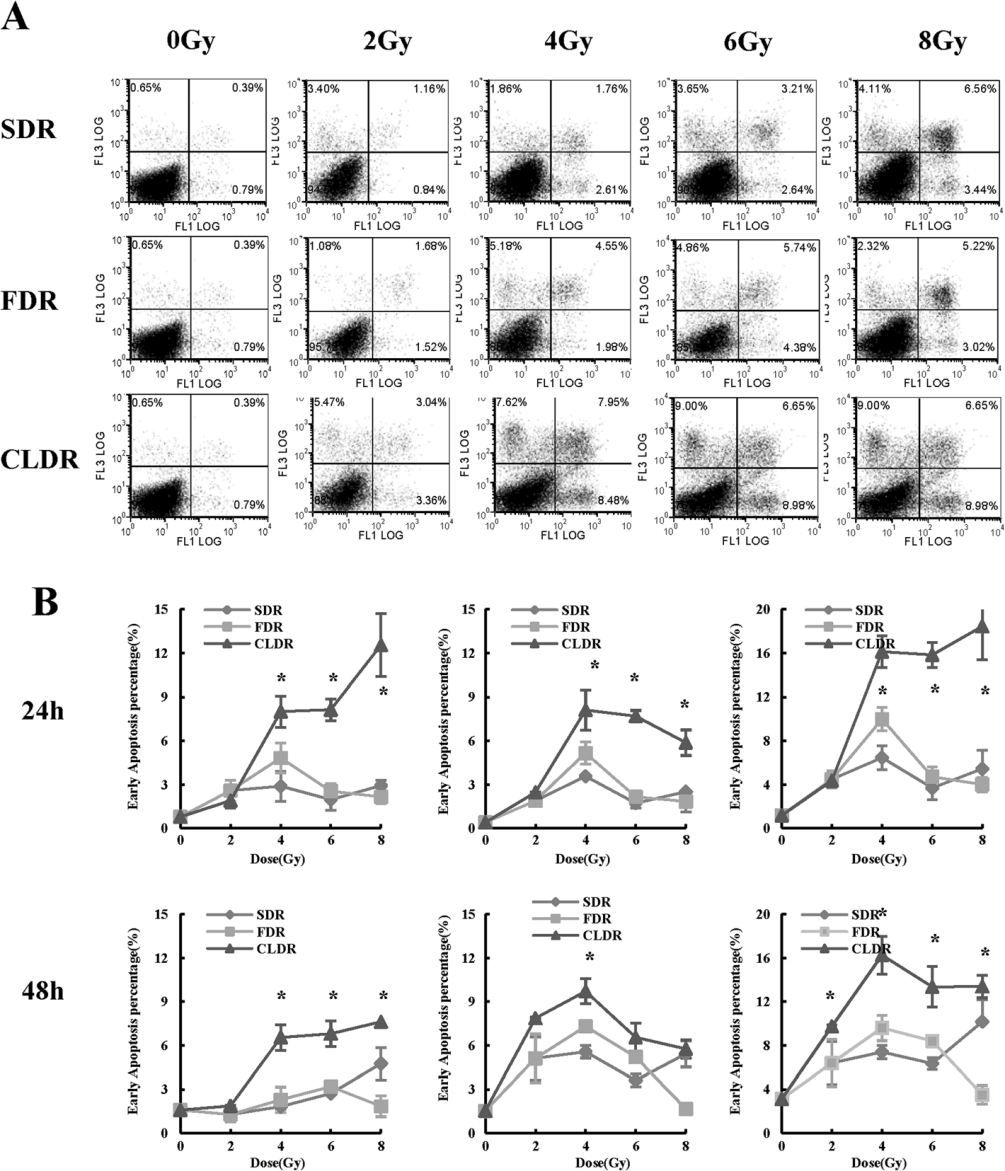
**Cell apoptotic assay of CL187 cells after distinct modes of irradiation. A**. In vitro assessment of apoptosis of CL187 cells. Percentage of apoptosis was measured by Annexin V and PI staining. The apoptosis was determined by FACS analysis of Annexin V-FITC and PI-staining 48 hrs after 0, 2, 4, 6 and 8Gy irradiation. Cells in the lower right quadrant indicate Annexin-positive, early apoptotic cells whereas the cells in the upper right quadrant indicate Annexin-positive/PI-positive, late apoptotic cells. **B**. Statistical analysis showed the percentages of early, late and total apoptosis of SDR, FDR and CLDR group 24, and 48 hrs after irradiation. The above data were one representative of three separate experiments, with three wells in each group. *p < 0.05. (show * in figures).

To further explore the biological effects of different modes of irradiation on CL187 cells, we examined the apoptotic rate upon irradiation. We found that CLDR resulted in increased levels of early and late apoptosis compared with SDR and FDR, especially 48 hrs after irradiation. As shown in Figure 5, early apoptotic rates of CL187 cells in CLDR group were 1.28 ± 0.25%, 1.87 ± 0.06%, 6.54 ± 0.88%, 6.80 ± 0.87% and 7.62 ± 0.39%, respectively, 48 hrs after 0, 2, 4, 6 and 8Gy irradiation. At this time point, the early apoptotic rate of CLDR group increased significantly compared with SDR and FDR group (CLDR vs. SDR, t = -31.22, p = 0.001; CLDR vs. FDR, t = -7.01, p = 0.02). Similarly, 48 hrs after 0, 2, 4, 6 and 8Gy irradiation, late apoptotic rates of CLDR group were 1.19 ± 0.05%, 7.86 ± 0.08%, 9.72 ± 0.85%, 6.56 ± 0.98% and 5.76 ± 0.63%, respectively, which showed a significant increase compared with SDR and FDR group (CLDR vs. SDR, t = -15.08, p = 0.004; CLDR vs. FDR, t = -11.99, p = 0.007).

### Impaired DNA repair capacity of CL187 cells upon CLDR

**Figure 6:**
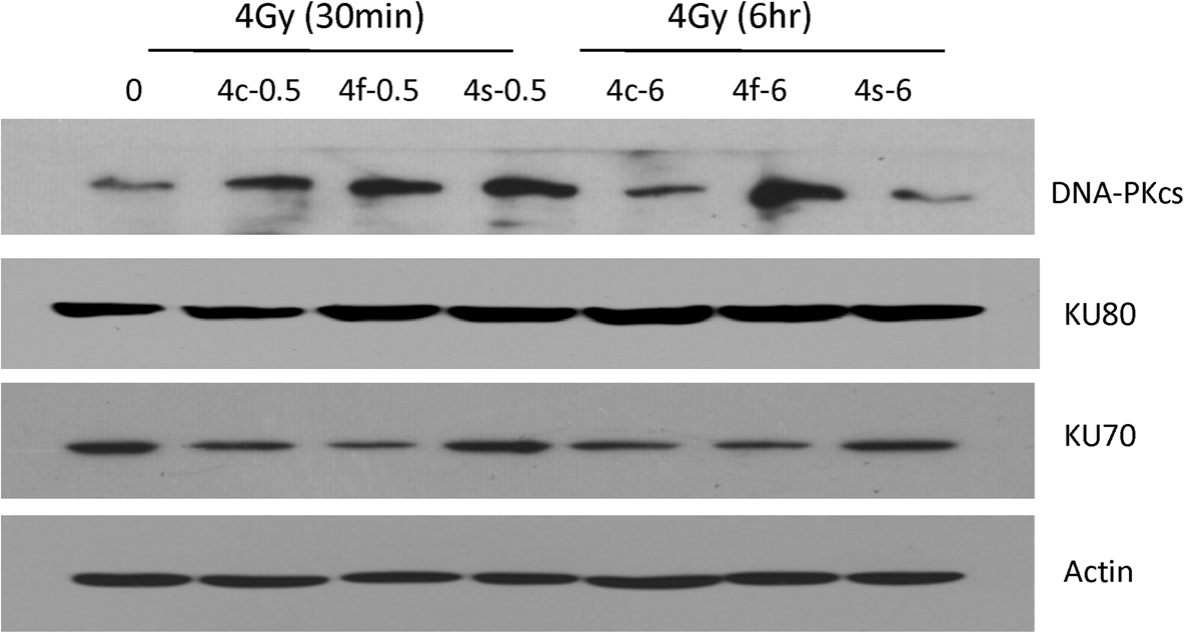
**CLDR down-regulated the expression level of DNA repair associated proteins.** Reduction of DNA repair proteins in CL187 cells after CLDR. Western blot analysis of DNA-PKcs, Ku70 and Ku80 expression level in CL187 cells 30 minutes or 6 hrs after 4Gy of SDR, FDR and CLDR. Beta-actin served as the internal control. One representative is shown from three separate experiments. 4c-0.5 represtnts the group of CLDR cells harvested 30 minutes after radiation, 4f-0.5 represtnts the group of FDR cells harvested 30 minutes after radiation, 4 s-0.5 represtnts the group of SDR cells harvested 30 minutes after radiation, 4c-6 represtnts the group of CLDR cells harvested 6 hrs after radiation, 4f-6 represtnts the group of FDR cells harvested 6 hrs after radiation, 4 s-6 represtnts the group of SDR cells harvested 6 hrs after radiation.

Ionizing radiation triggers several types of DNA damage including DNA single-strand breaks (SSBs) and DNA double-strand breaks (DSBs), and the latter results in cell death. Non-homologous end joining (NHEJ) is a crucial step responsible for repairing DNA-DSBs in mammalian cells, which is thus of great importance to maintain the survival of the irradiated cells. DNA dependent protein kinase (DNA-PK) complex is one of the major participants in NHEJ [15,16]. DNA-PK complex consists of two components: a 450-kd catalytic subunit DNA-PKcs and a hetero-dimeric protein Ku which is composed of two tightly associated but different polypeptides Ku70 and Ku80. Therefore, we determined the expression levels of DNA-PKcs, Ku70 and Ku80 by western blotting before and after different dose rates of irradiation, as shown in Figure 6. Interestingly, DNA-PKcs and Ku70 expression were down-regulated upon 4Gy CLDR treatment compared with SDR and FDR groups, while the expression of Ku80 is indistinguishable between each group, which suggest that DNA-PKcs and Ku70 may critically involved into the DNA repair procedure induced by CLDR.

## Discussion

Management of local–regional recurrence of rectal cancer after primary radical resection and post-operative radiotherapy remains challenging. There are few effective options for those patients, and the prognosis remains poor. Majority of patients could not receive re-operation, and some of them even could not receive re-irradiation. They are suffering unbearable symptoms such as continuous pain and bleeding, and their survival time decrease dramatically. ^125^I radioactive seed interstitial implantation provides an alternative choice for those patients. The clinical results are inspiring, but the underlying mechanisms are not quite clear [17,18]. Whether there are different radiobiological mechanisms between low and high dose rate irradiation is still undefined.

In the present study, we first investigated the killing effects of SDR, FDR and CLDR on CL187 cells in vitro. Compared with SDR and FDR, CLDR caused severe total cell number decrease, due to enhanced cell death. The reported RBE of ^125^I seed continuous low dose rate irradiation of different models and cell lines were among 1.2 and 2.1, and most of which were ranged from 1.3 to 1.5 [11,12,19-21]. Lehnert et al showed that ^125^I irradiation caused increased RIF-1 cell death, and the RBE for ^125^I compared with ^60^Co gamma rays was 1.5 [21]. Wang et al reported that cell apoptosis of PANC-1 cell line increased after CLDR, with RBE for ^125^I compared with ^60^Co gamma rays being 1.39 [11]. Liao et al demonstrated that CLDR resulted in increased PC-3 cell apoptosis, with RBE for ^125^I compared with ^60^Co gamma rays being 1.41 [12]. In the present study, we have shown that the RBE for ^125^I compared with 6 MV X- rays was 1.42, which is consistent with the previous data reported by our colleagues and others.

Impairment of cell cycle checkpoint control and disrupted balance of proliferation and apoptosis are very important attributes leading to cancer development and progression. G_2_/M arrest is one of the major characteristics of cancer cells after irradiation, which forces to slow down cell-cycle progression to repair DNA damage, otherwise damaged cell will be programmed to undergo apoptosis. Previous studies have suggested that CLDR mediated cancer inhibition mainly via induction of cell cycle arrest and apoptosis [22-25]. We found that CLDR triggered more G_2_/M phase arrest of CL187 cells and also induced increased level of early and late apoptosis.

The DNA damages caused by irradiation are positively correlated to the G_2_/M phase arrest. In this regard, we next decided to determine the expression levels of several important regulators of DNA damage. DNA DSBs caused by irradiation tend to impair the integrity of the genome and cause cell death, and agents regulating DNA repair progress were tend to affect radiosensitivity of CLDR [17,18]. We have known that the Ku70 and Ku80 heterodimer bind to free DNA ends at the DSB and thus recruit DNA-PKcs, to activate its kinase activity [26-28]. It has been well known that functional defects or absence of Ku70, Ku80 or DNA-PKcs subunit result in remarked deficiencies in DNA-DSB repair and thus are hypersensitive to ionizing radiation. Korabiowska et al found that loss of Ku70 and Ku80 expression occurs frequently in hereditary and sporadic colorectal tumors [29]. Mazzarelli et al demonstrated that Ku70 and Ku80 heterodimer expression associated with DNA end binding activity were significantly increased in human colorectal tumor tissue than normal tissue [30]. After studying 101 colorectal cancer patients, Komuro et al found that 52 patients had elevated Ku70 expression, which were related with depth of tumor invasion and prognosis of survival in the multivariate analysis [31]. It has been shown that the capacity of repairing damaged DNA varied according to the radiation dose rate. The increased frequency of cell apoptosis upon CLDR was, at least in part, due to the decreased expression level of DNA-PKcs and Ku proteins, which efficiently mediate the damaged DNA repair program caused by ionizing irradiation. However, the link connecting DNA repair program and cell cycle arrest as well as cell apoptosis is still missing. It will be interesting to investigate how the down-regulation of DNA-PKcs and Ku proteins occur in the context of CLDR.

In summary, our study provides a beneficial exploration of biological effects of CLDR. The induced G_2_/M arrest, increased cell death and the decreased DNA repair ability caused by CLDR may contribute to the enhanced inhibitory effects of CLDR on tumor cells, compared with SDR and FDR. Though several questions remained to be addressed, we believe that with development of fundamental research and accumulation of clinical experiences, ^125^I radioactive seed permanent implantation can be further transformed and properly used in malignant tumor therapy.

## Competing interests

The authors declare that they have no competing interests.

## Authors’ contributions

Hao Wang and Jinna LI carried out the process of cell culture and cell irradiation, Hao Wang, Jinna LI and Ang Qu carried out the molecular genetic studies, Hao Wang and Jingjia Liu did statistical analysis, Hao Wang, Yong Zhao and Junjie Wang participated in the sequence alignment and drafted the manuscript. All authors read and approved the final manuscript.
